# Supplementary material for: Rising tension in the Himalayas: A geospatial analysis of Chinese border incursions into India
Source: PLoS One. 2022 Nov 10;17(11):e0274999. doi: 10.1371/journal.pone.0274999 (PMC9648716; doi:10.1371/journal.pone.0274999)
Supplement: Attachment — Submitted filename: Review_comments.pdf [file pone.0274999.s002.pdf]

Title: **Rising tension in the Himalayas: a geospatial analysis of Chinese border incursions into India**

Manuscript Number: **PONE-D-22-04496**

The manuscript has attempted to understand the spatial dynamics of the Chinese border incursions into India along the Himalayas by employing statistical methods and game theory. The paper has done a satisfactory job, and maybe accepted for publication. A few minor revisions are suggested.

Abstract

- There is mention that incursions in the west sector precede incursions in the eastern sector, and vice-versa. Kindly clarify the usage of this statement since the paper does not give an appropriate reason for the same, and the statistical test reveals that incursions in the west and east are independent of one another, thus, rendering this statement contradictory.

Introduction

- Provide an appropriate citation and reference for the claims made in Lines 1 to 5.
- Lines 18-20 mention about a surplus that India enjoyed. Kindly clarify the nature of the surplus, economic, or political, etc.

Methods

- Line 66 mentions journalistic sources as a data source. Journalistic sources tend to be influenced by political bias, and thus can be unreliable. Kindly clarify the usage of sources. Furthermore, mention all the data sources as citations or as a separate section.
- Line 86 “News often seems….” Maybe omitted due to redundancy.
- Table 2 highlights the incursions in the western sector. Kindly, provide a table for the eastern sector too.
- The section beginning on Line 130, “Geographical, political….” maybe shortened considerably and shifted to the “Introduction” section, since the usage of these factors in the dataset seems unclear. Also, for claims made in this section, such as the one from Line 135-151, appropriate citations need to be provided.
- Line 288, mention clearly what is a “Colonel Blotto game”.

Results

- Rename the section as “Results and Discussion” since the results have been discussed under this heading only.
- Shift Lines 261-270 to the Methods section and mention under assumptions considered.
