# Supplementary material for: Rising tension in the Himalayas: A geospatial analysis of Chinese border incursions into India
Source: PLoS One. 2022 Nov 10;17(11):e0274999. doi: 10.1371/journal.pone.0274999 (PMC9648716; doi:10.1371/journal.pone.0274999)
Supplement: Attachment — Submitted filename: Response to reviewers.docx [file pone.0274999.s003.docx]

# The remarks and suggestions of the referees were very helpful to improve the content and presentation of the paper. We extended our statistical analysis and rewrote large parts of it. The following has been done

# We went through our data set and checked all entries once more in Lexis Nexis. Eight entries were removed and fourteen new entries were added. The statistical analysis has been redone.

# We split the data set into primary incursions and triggered incursions in an attempt to reduce the self-reinforcing nature of media attention.

# We added a k-means cluster analysis

# We added an ARIMA model of yearly primary incursions

# We took meteorological conditions into account

# Reviewer #1

The manuscript has attempted to understand the spatial dynamics of the Chinese border incursions into India along the Himalayas by employing statistical methods and game theory. The paper has done a satisfactory job, and maybe accepted for publication. A few minor revisions are suggested.

# Abstract

•	There is mention that incursions in the west sector precede incursions in the eastern sector, and vice-versa. Kindly clarify the usage of this statement since the paper does not give an appropriate reason for the same, and the statistical test reveals that incursions in the west and east are independent of one another, thus, rendering this statement contradictory.

Our statement was imprecise and should have been that local incidents precede a rising tension all along the LAC. We have rewritten the abstract to clarify this.

# Introduction

•	Provide an appropriate citation and reference for the claims made in Lines 1 to 5.

We added a reference for the reported number of casualties. Multiple media outlets reported on the incident, but we only refer to the report by the BBC.

•	Lines 18-20 mention about a surplus that India enjoyed. Kindly clarify the nature of the surplus, economic, or political, etc.

This issue was also raised by reviewer#2 and we now specified that it is a trade surplus. The exact numbers are in [46].

# Methods

•	Line 66 mentions journalistic sources as a data source. Journalistic sources tend to be influenced by political bias, and thus can be unreliable. Kindly clarify the usage of sources. Furthermore, mention all the data sources as citations or as a separate section.

Indeed. Journalistic sources are biased. We found that in 2019, journalists underreported the number of incursions for political reasons. We used the India government numbers as a benchmark for our data. Also, in the new version of the paper we now differentiate between primary and triggered incursions, in order to adjust (to some extent) for this. The reviewer suggests that we add the data as a separate section or appendix. We have made our data available now.

•	Line 86 “News often seems….” Maybe omitted due to redundancy.

Indeed. Omitted.

•	Table 2 highlights the incursions in the western sector. Kindly, provide a table for the eastern sector too.

Is now included in the table.

•	The section beginning on Line 130, “Geographical, political….” maybe shortened considerably and shifted to the “Introduction” section, since the usage of these factors in the dataset seems unclear. Also, for claims made in this section, such as the one from Line 135-151, appropriate citations need to be provided.

We shortened it from 81 lines to 50 lines, added references, and shifted this part to the introduction section.

•	Line 288, mention clearly what is a “Colonel Blotto game”.

We have described the game in more detail and we have also extended the statistical analysis of the PLA’s strategy.

# Results

•	Rename the section as “Results and Discussion” since the results have been discussed under this heading only.

Done

•	Shift Lines 261-270 to the Methods section and mention under assumptions considered.

We have now divided our data into primary and triggered incursions. This has been very helpful for our analysis. We shifted the description to the methods section.

Reviewer #2

Evaluation:

The analysis makes some interesting points and the data is new, but there are a number of issues. It is not clear how this data relates to other standard conflict datasets, the theoretical treatment of the literature on Blotto games and systems defense does not accurately represent what theory tell us about incentives related to this application, and the statistical analysis is not what would be considered the current standard in empirical conflict studies. It is possible all of these issue could be addressed in revisions, though I think because there are so few observations in the data it will be difficult to make many conditional statements about the effect of the variables of interest on incursion strategies they want to claim. But obviously that is an empirical question.

We have extended our statistical analysis in a way that is more up to standard. We have also made our data available. It contains 120 incursions based on media reports (ACLED contains 5 incidents, of the major military clashes).

I am also a little skeptical that we can conclude that the Chinese incursions in the west are strategically planned and aim for a permanent control, or at least a clear status quo of the contested areas from this analysis. I think we can conclude that maybe that is true, maybe asymmetric valuations are the cause, or asymmetric budget constraints. We can conclude that the targeting is not consistent with the incentives of a symmetric zero-sum military competition for territory.

We agree that we should have been more careful with drawing firm conclusions here. We revised and extended our analysis and have rewritten this part of the paper.

Other Comments:

p.2 line 19 Surplus in what?

Trade surplus. Rev#1 raised the same question.

What are the statistical implications for the selections of observations, i.e., China only and only those verified by multiple sources? What happens to the results if you include the unverified reports or Indian incursions?

This is of course a difficult issue. We went through lexisnexis again and deleted 8 incursions as dubious and added 14 new entries. The results remain practically the same.

In the symmetric Blotto game uniform randomization is the equilibrium strategy, with asymmetric values and resources, the marginal distributions are more complicated. It is not clear to me how these tests speak to the equilibrium of that game under the conditions of this problem.

In the asymmetric game the optimal strategy is practically incomputable. The players divide resources in some (hard to compute) way and assign uniformly randomly over the battlefields. It is hard to come up with precise numbers, but qualitatively, the weaker player chooses more extreme allocations and targets a limited number of battlefields. We tested for that. If the game is played over rounds, then the allocations should average out. We tested for that as well and we draw qualitative conclusions.

Doesn’t local geography and weather matter for the rates of incursions in the west and east? I was expecting some sort of conditional analysis (i.e., with controls related to target location). The basic thought experiment I have in mind is: suppose that China wanted to attack in the east and west every day, but can only do so when the weather is good. The daily weather is random and uncorrelated at the two locations, then the unconditional observation of incursions would be uncorrelated, even though, in fact, they are the product of the same perfectly correlated strategy: attack every day.

This is a very interesting suggestion and we checked the meteorological conditions in the red-zones on the days of incursions. We found that these conditions do not seem to play a role in the selection of the red-zone. It is not true that zone A is chosen over zone B because the weather is nicer in A. Since we found no significant results, we kept the discussion very brief, but we can extend on this and include this data, if necessary.

The description of the data is not as clear as it probably should be.

Reviewer #1 raises the same point and more strongly. We made our data available. It could be added as an appendix.

I don’t understand this claim: “The border dispute can therefore be divided into two separate conflicts, centered around Aksai Chin and Arunachal Pradesh.” The whole point of the NE in the Blotto game is that the conflicts are connected, but the strategic interaction (at least in the symmetric case) induces strategies that are uniform randomizations.

We only apply the Blotto game to the conflict in the western sector, where red-zones are relatively close. We found that incursions in west and east are statistically independent but the overall tension along the LAC is in sync.

When we got the periodicity analysis, I was expecting some kind of time series statistics, lagged regression analysis, or something. It is not clear to me exactly what the authors did here (It looks like simply doing correlations between y_t and y_{t-k} for some k’s), but it is not what I would consider standard practice to analyze time dependence in panel data.

Our time-series of incursions has only 15 years and is not trend stationary, because of the escalation of the conflict since 2013. This is why we avoided a time series analysis and only looked at cycles in the series. In our revision we now consider primary incursions, for which the numbers increase less sharply, and apply a lag regression to demonstrate that the cyclicity of the conflict will persist.
